# Long-term fiscal implications of funding assisted reproduction: a generational accounting model for Spain

**DOI:** 10.1016/j.rbms.2016.04.001

**Published:** 2016-05-13

**Authors:** R. Matorras, R. Villoro, A. González-Domínguez, S. Pérez-Camarero, A. Hidalgo-Vega, C. Polanco

**Affiliations:** a Human Reproduction Unit, Department of Obstetrics and Gynecology, Hospital de Cruces, Baracaldo, Vizcaya, Spain; b Department of Medical Specialities, University of the Basque Country, Leioa, Vizcaya, Spain; c Valencian Institute of Infertility, Bilbao, Spain; d Instituto Max Weber, Madrid, Spain; e Health Economics Department, Castilla La Mancha University, Spain; f Health Economics, Business Excellence and Corporate Development, Merck S.L., Spain^1^1 An affiliate of Merck KGaA, Darmstadt, Germany.

**Keywords:** generational accounting, health economics, health investment, IVF, live birth

## Abstract

The aim of this study was to assess the lifetime economic benefits of assisted reproduction in Spain by calculating the return on this investment. We developed a generational accounting model that simulates the flow of taxes paid by the individual, minus direct government transfers received over the individual’s lifetime. The difference between discounted transfers and taxes minus the cost of either IVF or artificial insemination (AI) equals the net fiscal contribution (NFC) of a child conceived through assisted reproduction. We conducted sensitivity analysis to test the robustness of our results under various macroeconomic scenarios. A child conceived through assisted reproduction would contribute €370,482 in net taxes to the Spanish Treasury and would receive €275,972 in transfers over their lifetime. Taking into account that only 75% of assisted reproduction pregnancies are successful, the NFC was estimated at €66,709 for IVF-conceived children and €67,253 for AI-conceived children. The return on investment for each euro invested was €15.98 for IVF and €18.53 for AI. The long-term NFC of a child conceived through assisted reproduction could range from €466,379 to €-9,529 (IVF) and from €466,923 to €-8,985 (AI). The return on investment would vary between €-2.28 and €111.75 (IVF), and €-2.48 and €128.66 (AI) for each euro invested. The break-even point at which the financial position would begin to favour the Spanish Treasury ranges between 29 and 41 years of age. Investment in assisted reproductive techniques may lead to positive discounted future fiscal revenue, notwithstanding its beneficial psychological effect for infertile couples in Spain.

## Introduction

Birth and fertility rates in Spain have declined significantly in recent years. In 2011 the birth rate stood at 10.2 births per 1000 inhabitants, its lowest level since 2003, while the fertility rate was 1.36 children per woman (INE National Institute of Statistics, 2014), which is below the replacement level (2.1). This is partly attributable to the greater integration of women into the labour market and consequent delay in maternity (IMW Instituto Max Weber, 2012), but it is also compounded by the prevalence of infertility, defined as the inability of one or both partners to conceive naturally after a year of frequent unprotected sexual intercourse. Infertility affects 15% of the population in Spain, or one in seven couples of reproductive age (Matorras Weinig, 2011). Currently, the Spanish public health system offers two types of assisted reproduction: IVF and artificial insemination (AI). In both, the public health system finances a maximum of three IVF cycles or four AI cycles, or a combination of both techniques, whether pregnancy is achieved or not. Inclusion criteria for receiving publicly financed assisted reproduction are women between 18 and 40 years of age, who are physically and mentally fit (no definition provided), who have been classified as infertile according to the previous definition but have no evidence of a poor ovarian reserve. Priority is given to nulliparous and infertile couples (Alberto et al., 2002). In 2010, there were approximately one million couples requesting assisted reproductive treatment. Only 22% received one or more assisted reproductive treatment cycles, of which 65% were performed in private facilities. The average waiting time for an AI or IVF cycle in a public health facility was 339 days (Matorras Weinig, 2011), so women or couples who can afford to pay for treatment, and those who do not fulfil inclusion criteria, may choose to go to a private health facility without a waiting list (Matorras Weinig, 2011). Therefore, not only is there an excess demand for assisted reproductive treatment but also inequity of access to that treatment.

Previous studies have shown that, even under the most optimistic projections, a declining birth rate combined with an ageing population might result in fiscal imbalances that can only be mitigated through spending cuts or tax increases (IMW Instituto Max Weber, 2012, MESS Ministry of Employment, 2006, National Research Council, 2012, Pérez-Camarero et al., 2012). A higher birth rate would contribute to a medium-term increase in the taxpaying population and offset the imbalances caused by the growth of the ageing population.

In this context, public funding of assisted reproductive treatment could have a positive effect not only on mitigating the excess demand and the current inequity in access to healthcare services, but it could help restore replacement rates and improve fiscal stability (Pérez-Camarero et al., 2012). Indeed, beyond the obvious benefit of assisted reprodution allowing a couple to conceive, generally improving their quality of life, it could produce a social benefit that goes far beyond the direct individual benefit, because each individual conceived contributes a tax flow to society, especially during his/her working life. Since the individual also ‘costs’ society the amount of pensions and public services he/she receives, the net benefit that an individual brings to society is quantifiable as the taxes paid minus benefits received.

The long-term fiscal implications of public funding of assisted reproductive treatment have been estimated previously in countries as diverse as Brazil (Kröger and Ejzenberg, 2012), Denmark (Connolly et al., 2011), Sweden (Svensson et al., 2008), the UK (Connolly et al., 2009), the USA (Connolly et al., 2008) and, more recently, Greece (Fragoulakis and Maniadakis, 2013), The Netherlands (Moolenaar et al., 2014), Ukraine, Belarus and Kazakhstan (Mandrik et al., 2015). These studies use generational accounting models that estimate the social net benefit of an IVF-conceived individual. With the exception of the Dutch case, they conclude that such funding would bring net fiscal benefits to the State over the lifetime of the individual. However, these results are not directly transferable to Spain, since they apply only within the context of each country’s own fiscal, healthcare and welfare policies.

In order to investigate whether funding assisted reproduction might be considered an efficient use of public resources in Spain, this analysis models the long-term tax implications that public funding of assisted reproduction might have in the country. Using a generational accounting model, we analyse the fiscal balance of the economic transfers between the government and an individual conceived through assisted reproductive treatment over his/her lifetime. Both IVF and AI treatments are considered.

## Materials and methods

A generational accounting model was built to estimate the net fiscal value of an individual conceived through assisted reproductive treatment (Auerbach et al., 1991, Auerbach et al., 1992, Auerbach et al., 1994, Cardarelli et al., 2000). To analyse the fiscal relationship between the State and an individual throughout his/her life cycle the model calculates the sum of all gross income that the State will receive from the individual, minus direct transfer expenses throughout his/her lifetime. The net present value (NPV) of future flows of revenues and transfers for each individual is as follows:

(1) $$\mathrm{NPV}=\sum_{t=0}^{N}\left[\frac{T_{t}-X_{t}}{{\left(1+r\right)}^{t}}\right]-K_{0}$$

where *N* is the individual’s life expectancy, *T* is gross revenues received by the government through taxes paid by the individual, *X* is direct transfers to the individual (e.g. healthcare, education and pensions), *K* is the cost of assisted reproductive treatment, *r* is the discount rate and *t* corresponds to 1 year. Revenues and transfers are apportioned to each year of the individual’s life, from age 0 to the age of life expectancy. We assume that an individual conceived through assisted reproductive treatment is an average individual in terms of education, income, health status, life expectancy, probability of being unemployed and likelihood of being a public servant. Therefore, the model apportions transfers and taxes weighted by the probability of the individual belonging to any category of taxpayers or potential recipients (for example, the probability of being employed, of receiving education grants, of being a business owner, of being unemployed and receiving unemployment benefits in any year across his/her lifecycle). The model adopts the perspective of the Spanish government and includes the full official list of revenues collected by the State, which comprise not only taxes paid by individuals in the labour force, but also those paid by individuals regardless of age or employment status (e.g. VAT, taxes paid on tobacco, alcohol, hydrocarbons, etc.). Transfers to individuals include education, healthcare and pensions from birth to life expectancy, plus the average cost of IVF or AI cycles needed to achieve a successful pregnancy, which is assumed only once, in year zero of the model. For an overview of all revenues and expenditures, see Table 1.

**Table 1:** Overview of government revenues and expenditures.

*Revenue*	*Expenditure*
Income tax Social Security contributions Corporate tax Non-resident income tax Taxes on alcohol consumption Taxes on tobacco consumption Tax on petrol and diesel oil consumption Value-added tax Other taxes (electricity, insurances, sugar consumption, fiscal sanctions).	Cost of assisted reproduction Education Healthcare Pensions Governmental employees’ salaries Unemployment benefits

### Base year and time horizon

The base year is the hypothetical year of birth of the individual whose net tax value the model calculates. In the base case, the model assumes that the base year macroeconomic parameters, especially the rate of economic growth and the unemployment rate, are constant over the time horizon. In order to account for possible business cycles contractions and expansions that will occur during the lifetime of the individual, we conducted a linear regression of the annual growth rate of gross domestic product (GDP) in Spain for the period 1970–2012 (World Bank, 2013). In this period the average GDP growth rate was 4.09%. The year with the closest average GDP growth was 2006 with 4.08%, and this year was therefore set as the base case. All monetary units were converted into the equivalent of 2006 using the consumer price index (INE National Institute of Statistics, 2013).

The model’s time horizon is the individual’s life expectancy at birth in Spain in the base year (78 years), which is consistent with previously published empirical models (Connolly et al., 2008, Connolly et al., 2009, Connolly et al., 2011, Fragoulakis and Maniadakis, 2013, Kröger and Ejzenberg, 2012, Svensson et al., 2008). Table 2 lists the values assigned to the main parameters used in the base case scenario.

**Table 2:** Values assigned to the base case scenario.

*Parameter*	*Amount*
Annual discount rate (%)	3.5
Annual GDP growth rate (%)	4.08
Annual inflation rate (%)	3.5
Retirement age (years)	65
Unemployment rate (relative to the total population over 16 years) (%)	10.61
Percentage of public servants in the labour force (%)	7.81

### Discount rate and labour productivity growth

To reflect the depreciation of money over time, we applied a discount rate of 3.5% on all transfers to and from the government. This is consistent with discount rates used in previous studies, which range between 2.5% (Svensson et al., 2008) and 4.0% (Kröger and Ejzenberg, 2012, Moolenaar et al., 2014). To account for economic growth over time, costs were adjusted according to GDP growth rate in the base year (Auerbach et al., 1999, Cardarelli et al., 2000), which was 4.08% and an annual inflation rate of 3.5%.

### Cost of assisted reproduction

There are no official data available on the cost of assisted reproduction in the Spanish public health system. IVF and AI costs were based on two published studies that estimate direct healthcare costs of both IVF and AI cycles in two different Spanish hospitals (Matorras Weinig, 2011, Matorras and Valladolid, 2001, Navarro Espigares et al., 2006). An average success rate of 28.7% was used to calculate the cost per live birth (SEF Spanish Fertility Association, 2001). If the government finances only three assisted reproductive treatment cycles, the expected investment in assisted reproduction is €2661 for IVF, and €1424 for AI (2006 costs). The expected average cost of a pregnancy is €4173 for IVF and €3629 for AI. The fact that only 75% of all pregnancies result in a live birth is taken into account when modelling expected benefits and costs in the model. Delivery costs were not considered in the model because they are assumed to be similar between children conceived through assisted reproductive treatment and those conceived naturally.

### Transfers from the government to the individual

Transfers from the government to the individual were obtained from different sources. In most cases the data are available at the aggregate level, so we estimated what the average individual received on each item. When possible the average by age year is imputed. Otherwise, when data is available by age groups, the average by age group is imputed to each age year within the same age group. In the minority of cases, when age distributions are not available, an average per capita is imputed to all years. All data were obtained for 2006.

The sum of transfers from the government to the citizens at time *t* can be expressed as:

$$X_{t}=E_{\mathrm{it}}\left(pE_{\mathrm{it}}\right)+H_{t}\left(pH_{t}\right)+W_{t}\left(pW_{t}\right)+P_{\mathrm{it}}\left(pP_{\mathrm{it}}\right)+U_{t}\left(pU_{t}\right),$$

where *E* is education, *H* is healthcare, *W* is salaries of governmental employees, and *U* is unemployment benefits.

### Education

Education expenditure by schooling level (categories were: early childhood and primary education, middle school, high school, vocational training, and university) and through grants (non-university and university grants) was obtained from the Ministry of Education databases (MECD Ministry of Education, 2013). Enrolment rates by age and education level were obtained from the National Institute of Statistics (INE National Institute of Statistics, 2008). We calculated the average transfer by individual for each age and adjusted it to the proportion of individuals receiving the transfer.

### Healthcare

Public healthcare expenditure by age group (0–4, 5–14, 15–44, 45–64, 65–74, > 74 years) was obtained from the Ministry of Health (MSSSI Ministry of Health, 2011) for 2006. The population size of each of these groups is available from the Population Data Series (National Institute of Statistics, 2014). We assigned average expenditure by age group to each corresponding age of the individual.

### Pensions

Pensions received by individuals include orphanhood, widowhood and retirement pensions. The proportion of pensioners and the average amount in euros received in each pension category can be obtained by age (0–78) from the Spanish Labour Force Survey, which is representative at the national level and collects information at the individual level (MESS Ministry of Employment, 2006). We assigned average pensions to the corresponding age of the individual, from 0 years of age to 78.

### Salaries of governmental employees

This model includes the wages spent by the government on all governmental employees. Total wages were obtained from the (MEH Ministry of Finance, 2007). The distribution of governmental employees by age group (11 age groups from 16 to 85 years of age, grouping years into 5 year-bundles) is available from the National Institute of Statistics (INE National Institute of Statistics, 2007). We imputed the average wage by age group to each year in the life of the ‘individual’.

### Unemployment benefits

Average unemployment benefits and unemployment rates by age group (< 25, 26–35, 36–45, 46–55, > 55) are available from the Spanish Treasury databases (AT Treasury Statistics Databases, 2013). We imputed average benefits adjusted by the proportion of unemployed to each year within the same age group.

### Other transfers

Tax exceptions due to parenthood are implicitly included in the income tax declared by the individual, which is a transfer from the individuals to the government in the model. As opposed to other countries, child benefit payments do not exist in Spain, and data on paid leave due to parenthood are not available. These two parameters were therefore not included in the model.

### Transfers from the individual to the government

The sum of transfers received by the government from the individual at time *t* is:

$$T_{t}={\mathrm{SS}}_{t}+I_{\mathrm{it}}+C_{t}+N_{t}+A_{t}+H_{t}+{\mathrm{VAT}}_{t}+{\mathrm{Tob}}_{t}+O_{\mathrm{it}},$$

where *SS* stands for Social Security contributions, *I* is income tax, *C* is corporate tax, *N* is non-resident income tax, *A* is tax paid on alcohol and beer consumption, *H* is tax paid on petrol and diesel, *Tob* is tax paid on tobacco consumption, *VAT* is value added tax, and *O* refers to other transfers.

### Social Security contributions

In Spain all employed persons pay a Social Security contribution. Contributions are age- and employment regime-dependent. We used the Spanish Labour Survey (MESS Ministry of Employment, 2006), which collects information on Social Security contributions at the individual level to calculate the proportion of persons working by age and the average contribution by age. The average contribution was weighted according to the proportion of individuals working in each age year (0–78) and the result was imputed to each year of age of the hypothetical individual in the model.

### Income tax

This category includes the income tax paid by employed individuals in the private sector (including self-employed) and public sector employed individuals. Private sector income tax is available at the individual level from the Spanish Labour Force Survey (MESS Ministry of Employment, 2006). Income tax paid by the public sector working force is available at the aggregate level from the Treasury’s annual report (AT Treasury Annual Report, 2008). The number and proportion of public sector workers in 2006 is available from the National Institute of Statistics (INE National Institute of Statistics, 2007). We calculated average private sector income tax by age and category (employed and self-employed workers) and public sector income tax per public sector worker. We weighed these averages by the proportion of people working under each category (employed, self-employed and public sector worker) at each age using official employment statistics (INE National Institute of Statistics, 2007), and imputed the sum of weighted averages to each corresponding age in the model.

### Corporate tax

Corporate tax is paid by business owners, and is available at the aggregate level from the Treasury’s Annual Report (AT Treasury Annual Report, 2008). The number of business owners is available from official employment statistics (INE National Institute of Statistics, 2007) by 5-year age groups from age 16 onwards. Average tax per business owner was adjusted for the proportion of business owners in each age group.

### Non-resident income tax

This tax is paid by Spaniards living abroad. It is available at the aggregate level from the Treasury’s Annual Report (AT Treasury Annual Report, 2008). The number of non-residents is reflected in the National Register of Non-Resident Spaniards (INE National Institute of Statistics, 2009a, INE National Institute of Statistics, 2009b) by 5-year age groups from age 15 onwards. Average tax per non-resident was adjusted for the proportion of non-residents in each age group.

### Taxes on alcohol and tobacco consumption

The revenue from alcohol and beer, and tobacco consumption are available from the Spanish Treasury Annual Report (AT Treasury Annual Report, 2008) at the aggregate level. The proportion of alcohol consumers and of smokers are available from the Spanish data in the European Health Interview Survey (INE National Institute of Statistics, 2009a, INE National Institute of Statistics, 2009b). Spain joined this survey in 2009, so no data are available for 2006. We assumed that the proportion of the population who drink and smoke in 2006 were the same as in 2009. We calculated average tax per alcohol consumer and average tax per smoker and adjusted them by the proportion of alcohol consumers and smokers by age, respectively.

### Tax on petrol and diesel oil consumption

The revenue from the sale of petrol and diesel is available from the Spanish Treasury Annual Report (AT Treasury Annual Report, 2008) at the aggregate level. The Spanish Drivers Census (MIDGT Ministry of Interior, 2008) offers the number of persons who own a driver’s licence by 5-years age categories, from the age of 13. The average tax per driver was adjusted by the proportion of drivers in each age category.

### Value added tax

The revenue from VAT is available from the Spanish Treasury Annual Report (AT Treasury Annual Report, 2008) at the aggregate level. We calculated a per capita VAT from age 17.

### Other transfers from the individuals to the government

Other transfers include taxes paid for inheritance, electricity, insurances, sugar consumption, and fiscal sanctions. All are available from the Spanish Treasury Annual Report (AT Treasury Annual Report, 2008) at the aggregate level. We calculated a per capita taxes from age 18.

### Sensitivity analysis

Due to the uncertainty of the country’s economic growth, and to test the robustness of the model’s results in a scenario of economic crisis, one-way deterministic sensitivity analysis was performed. The analysis included eight different scenarios where we vary, either alone or combined, those parameters most likely to change in the future and to have an impact on the results: discount rate (1% and 5%), GDP growth rate (± 100%), inflation rate (± 100%), and unemployment rate (± 100%).

## Results

**Fig. 1:**
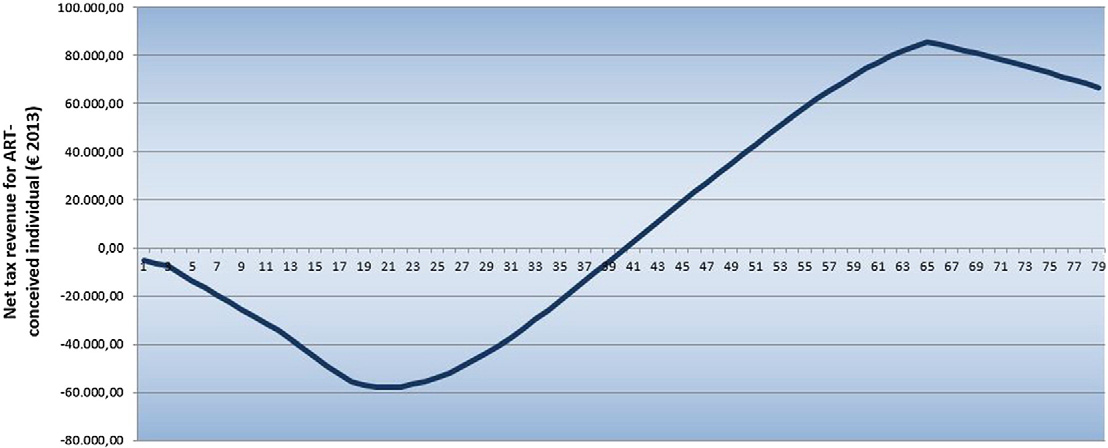
Cumulative individual net fiscal contribution: base case scenario. ART = assisted reproductive techniques.

Fig. 1 shows the cumulative discounted net fiscal contribution of an average individual conceived through assisted reproductive treatment with IVF. Investment is done in year zero, which explains why the first value of the curve is negative. During the first years of life, children are net recipients of public transfers, which, from the government perspective, represents a cumulative deficit between birth and 18 years. At this age, the curve reaches a turning point: taxes paid by the individual, either through consumption or work, are higher than what he/she receives for education, healthcare, unemployment or other benefits. At approximately age 39, the state revenues and accrued expenses come into balance. From that age, an average individual starts to contribute more in taxes than he/she receives in transfers until retirement age. During retirement, individuals typically consume more public benefits, such as pensions and healthcare, compared with their consumption over their working life. This explains the negative slope of the curve drawn from retirement age to 78 years, the life expectancy of the individual.

Table 3 shows the results obtained in the base case scenario for a naturally conceived individual and an individual conceived through either IVF or AI, if the government were to fully fund all cycles required to obtain a pregnancy, and if it were to finance only three cycles of either assisted reproductive technique, as it currently does in Spain. These results take into account that only 75% of pregnancies conceived through assisted reproduction result in a live birth and present the revenues raised through taxes, state transfers to individuals (including the cost of IVF or AI), the NFC of the individual in present value, with and without assisted reproduction, the break-even age point, which is the age at which state expenses (including expenditure on assisted reproduction) equal revenues, and the return on investment for every euro spent on assisted reproduction in year zero.

**Table 3:** Results of investing on an assisted reproduction pregnancy and on three assisted reproductive cycles: base case scenario.

		*As many cycles as needed to obtain one pregnancy*^a^	*Three cycles*
Naturally conceived individual	Taxes received (€)	370,482	
	Transfers to individual (€)	275,972	
	NFC (€)	94,510	
IVF-conceived individual^b^	Expected NFC (€)	70,883	45,190
	Expected NFC minus IVF cost (€)	66,709	42,529
	Break-even age (years)	39	39
	Return on investment (€)	15.98	15.98
AI-conceived individual^c^	Expected NFC (€)	70,883	27,811
	Expected NFC minus AI cost (€)	67,253	26,387
	Break-even age	39	39
	Return on investment (€)	18.53	18.53

a Probability of successful pregnancy is 0.75.

b Number of cycles needed for pregnancy = 3.48.

c Number of cycles needed for pregnancy = 6.53.

In the base case, the present value of all taxes paid by a naturally conceived individual equals €370,482, while the transfers made to the individual add up to €275,972. Therefore, the NFC of a naturally conceived individual throughout his/her lifetime is €94,510. In year zero the cost of achieving a live birth through IVF (taking into account the total number of IVF cycles needed for conception and the probability of the pregnancy resulting in a live birth) is €4173 while that of AI is €3629. Therefore, in the base case scenario, an individual conceived through assisted reproduction will have a NFC over his/her life cycle of €66,709 if conceived through IVF and of €67,253 if conceived through AI. These results imply returns on investment of €15.98 and €18.53 for every euro invested in IVF and AI, respectively.

If the government were to finance only the first three cycles of either assisted reproductive technique (as it does now) as opposed to financing the necessary cycles to reach a pregnancy, the expected cost of a successful pregnancy would equal €2661 if conceived through IVF and €1424 if conceived through AI, and the net fiscal contributions would be €42,528 for an individual conceived through IVF and €26,387 for an individual conceived through AI, which imply returns on investment of €15.98 and €18.53, respectively. The return on investment does not vary depending on whether the government decides to finance only three cycles or the total number of cycles required for conception, because the expected benefits increase in the same proportion as the expected costs.

### Sensitivity analysis

**Fig. 2:**
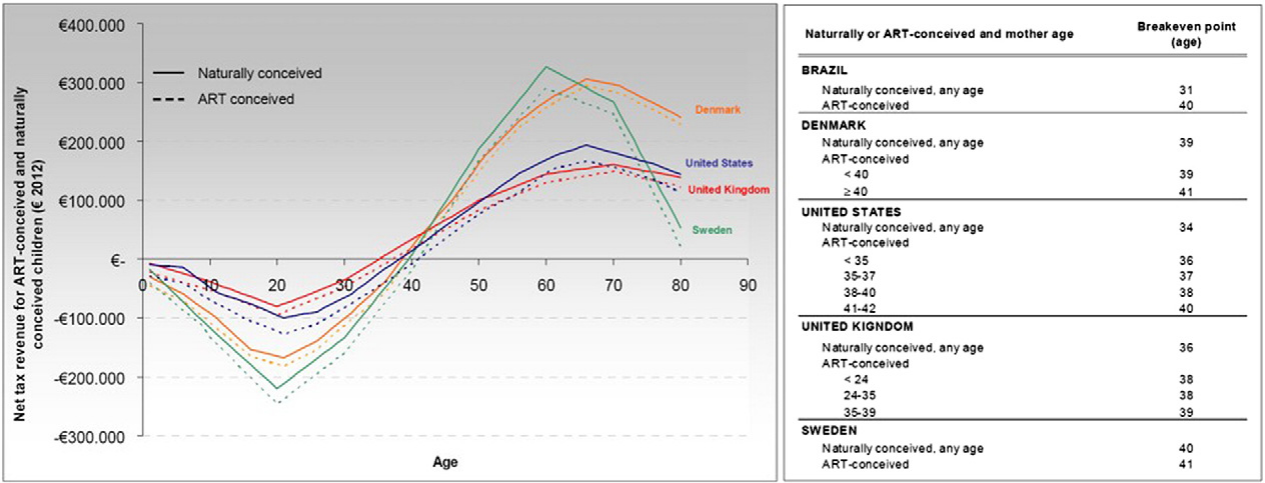
Lifetime net tax contributions and break-even ages for individuals conceived through assisted reproduction in previous analyses (Connolly et al., 2008, Kröger and Ejzenberg, 2012). ART = assisted reproductive techniques.

Table 4 shows the results obtained in the sensitivity analysis for an individual conceived through either IVF or AI, if the government were to fully fund all cycles required to obtain a pregnancy. In this case, the NFC of an IVF-conceived individual over her lifetime would vary between €466,379 (scenario 8, which assumes that the GDP growth rate doubles, inflation rate doubles, and unemployment rate is equal to zero) and €-9529 (scenario 7, which assumes a GDP growth rate equal to zero, an inflation rate equal to zero, and an unemployment rate that doubles the one in the base case). The NFC of an AI-conceived individual over his/her life cycle would range from €466,923 to €-8985, under the same scenarios. The age at which expenses and revenues balance out in individuals conceived through assisted reproduction ranges from 29 years to 41 years, which is similar to the break-even age in previous studies: USA, 34 years (Connolly et al., 2008); Brazil, 40 years (Kröger and Ejzenberg, 2012); Denmark, 39 years (Connolly et al., 2011); UK, 36 years (Connolly et al., 2009); and Sweden, 41 years (Svensson et al., 2008). See Fig. 2. Table 5 shows the results obtained in the base case scenario and the sensitivity analysis for a an individual conceived through ART if the government were to finance only three cycles of either ART technique, as it currently does in Spain.

**Table 4:** Results of investing on an assisted reproduction pregnancy: sensitivity analysis.

*Scenario*	*Varied parameters(%)*^a^	*IVF-conceived individual*^b^			*AI-conceived individual*^c^		
		*Expected NFC*^d^*(€)*	*Expected NFC minus IVF cost (€)*	*Break-even age (years)*	*Expected NFC*^d^*(€)*	*Expected NFC minus AI cost (€)*	*Break-even age (years)*
1	g -100; i -100	968.28	-3,205.13	39	968.28	-2,660.94	39
2	g + 100; i + 100	416,613.38	412,439.96	30	416,613.38	412,984.16	30
3	d = 1	249,807.82	245,634.40	30	249,807.82	246,178.60	30
4	d = 5	24,498.09	20,324.67	37	24,498.09	20,868.87	37
5	U -100	82,594.64	78,421.23	33	82,594.64	78,965.42	33
6	U + 100	43,368.84	39,195.43	35	43,368.84	39,739.63	35
7	g -100; i -100; U + 100	-5,355.40	-9,528.81	41	-5355.40	-8,984.61	41
8	g + 100; i + 100; U -100	470,552.39	466,378.97	29	470,552.39	466,923.17	29

a g = GDP growth rate; I = inflation rate; d = discount rate; U = unemployment rate. -100% indicates that the parameter becomes zero and + 100% indicates that the original value of the parameter is doubled.

b Number of cycles needed for pregnancy = 3.48.

c Number of cycles needed for pregnancy = 6.53.

d Probability of successful pregnancy = 0.75.

**Table 5:** Results of investing on three assisted reproduction cycles: sensitivity analysis.

*Scenario*	*Varied parameters (%)*^a^	*IVF-conceived individual*^b^			*AI-conceived individual*^c^		
		*Expected NFC*^d^*(€)*	*Expected NFC minus IVF cost (€)*	*Break-even age (years)*	*Expected NFC*^d^*(€)*	*Expected NFC minus AI cost (€)*	*Break-even age (years)*
1	g -100; i -100	617.31	-2,043.38	39	379.91	-1,044.03	39
2	g + 100; i + 100	265,604.74	262,944.05	30	163,460.17	162,036.23	30
3	d = 1	159,260.70	156,600.01	30	98,013.24	96,589.30	30
4	d = 5	15,618.34	12,957.65	37	9,611.94	8,188.00	37
5	U -100	52,656.80	49,996.11	33	32,406.38	30,982.45	33
6	U + 100	27,649.07	24,988.38	35	17,015.96	15,592.03	35
7	g -100; i -100; U + 100	-3,414.24	-6074.93	41	-2,101.21	-3,525.15	41
8	g + 100; i + 100; U -100	299,992.63	297,331.94	29	184,623.38	183,199.44	29

a g = GDP growth rate; I = inflation rate; d = discount rate; U = unemployment rate. -100% indicates that the parameter becomes zero and + 100% indicates that the original value of the parameter is doubled.

b Number of cycles needed for pregnancy = 3.48.

c Number of cycles needed for pregnancy = 6.53.

d Probability of successful pregnancy = 0.75.

**Fig. 3:**
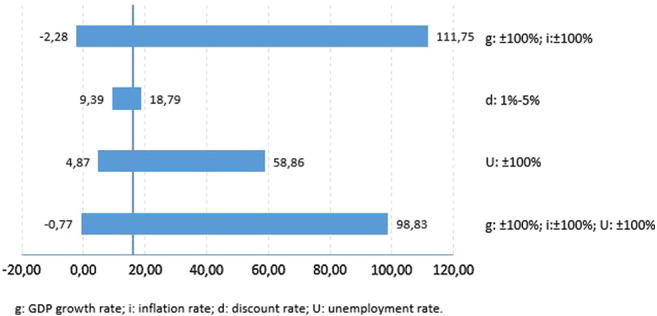
Tornado diagram: the return on investment of an IVF pregnancy.

**Fig. 4:**
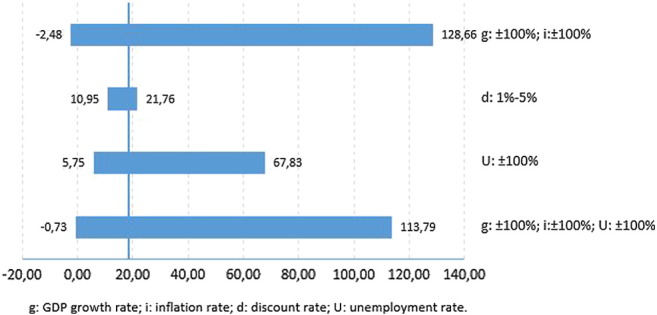
Tornado diagram: the return on investment of an AI pregnancy.

Fig. 3, Fig. 4 show the return on investment obtained in the sensitivity analysis for an individual conceived through either IVF or AI, respectively, if the government were to fund fully all cycles required to obtain a pregnancy. The return on investment per individual conceived through assisted reproduction ranges from €111.75 to €-2.28 for IVF and from €128.66 to €-2.48 for AI.

## Discussion

This is the first empirical study that estimates the NFC of an individual conceived through assisted reproduction in Spain. It is also the first study to compare IVF with AI techniques. Previous studies that have calculated the NFC for the life cycle of an IVF-conceived individual have shown that this can vary, but that, with the exception of The Netherlands (Moolenaar et al., 2014), it remains positive even in economic crisis scenarios: USD 61,428 in Brazil (Kröger and Ejzenberg, 2012); GBP 109,939 in the UK (Connolly et al., 2009); USD 155,870 in the USA (Connolly et al., 2008); SEK 254,000 in Sweden (Svensson et al., 2008); €154,100 in Denmark (Connolly et al., 2011); and €60,435 in Greece (Fragoulakis and Maniadakis, 2013). Our study indicates that public funding of assisted reproductive techniques is likely to result in a positive fiscal return for the Spanish Treasury. In the base case scenario, an IVF-conceived individual would have an NFC of €66,709, and each euro invested in assisted reproduction results in tax benefits of €15.98 to the State, while these figures are €67,253 and€18.53 in the case of AI-conceived individuals, which implies that AI represents a better investment compared with IVF. The NFC by an individual conceived through assisted reproduction remains positive in almost every circumstance in the sensitivity analysis for both techniques and also in the case where the government were to fund only three cycles of either technique, as is currently the case in Spain.

Our model is significantly more accurate than most previous empirical models for several reasons. First, previous empirical models assume that an individual’s economic life cycle can be divided into specific periods depending on his/her age (for example: ‘childhood’ (0–19 years), ‘working years’ (20–64 years), ‘senior years’ (65 + years)) in which the same average transfers and taxes are apportioned for all individuals belonging to the same periods, and regardless of exact age, true schooling level or working status (Connolly et al., 2008, Connolly et al., 2009, Connolly et al., 2011, Fragoulakis and Maniadakis, 2013, Kröger and Ejzenberg, 2012, Svensson et al., 2008). In our model, Treasury revenue and expenditure are apportioned in each year of the individual’s age, from age 0 to the age of life expectancy (78 years in this case) as weighted averages due to the probability (based on official statistical proportions) of belonging to a particular social group such as student, grant recipient, unemployed, public servant, pensioner, etc. In this way, in our model the ‘average’ individual represents all sectors of society. Second, unlike other studies where all taxes charged are generally limited to income tax and value added tax per capita, our data sources allow us to include the full official list of individual taxes collected by the Spanish Treasury, including not only taxes paid by individuals in the labour force, but also those paid by individuals regardless of age or employment status (e.g. taxes paid on tobacco, alcohol, petrol, etc.).

Of course, this model, like all mathematical representations of real-life situations, also has limitations. First, the model estimates the tax benefits that public funding of assisted reproduction would have in women up to 40 years of age. Although performing assisted reproductive techniques in women over 40 years of age has lower associated rates of success, studies in other countries also obtain positive – though less substantial – NFC for individuals born to women over 40. In Spain the public health system finances assisted reproduction only for women up to 40 years of age, a restriction that influences our data. We believe it would be beneficial to carry out a study that would include the costs of financing pregnancies in women over 40 years, to see whether public resources would be well invested in age ranges above 40.

Second, it is important to consider that 20% of IVF pregnancies are multiple (Sunderam et al., 2012). Data on the incidence of multiple pregnancies in Spain is not available, but it is sensible to forecast that with more than one individual born after assisted reproduction funding, the return on investment would be multiplied by the number of children produced. Moreover, many individuals conceived through assisted reproduction eventually have children of their own, who in turn might contribute a positive NFC to the government. On the other hand, the risk of premature labour in multiple pregnancies is greater than in single births (Kurdi et al., 2004, Pandian et al., 2005). Although no data are available for Spain, in the literature this risk ranges between 42% and 33.4%, versus 12.2% and 6.4% in single births (Janvier et al., 2011, Kurdi et al., 2004). Fifty per cent of premature births have prenatal complications (Janvier et al., 2011). If funding of assisted reproduction increases in Spain, further funding will probably be necessary to cover the extra costs associated with premature births. Our model does not include the additional healthcare costs associated with the care of premature infants with complications. The inclusion of this data would enrich the accuracy of the results.

Third, since this analysis is performed from the perspective of the Spanish Treasury, our results do not include the estimated individual benefits in terms of quality of life that conceiving provides for couples who suffer from infertility, even extending to the ‘peace of mind’ experienced by those couples who should they fail to conceive, know that they have tried everything. Inclusion of the psychological and social benefits of artificial conception would reveal even greater advantages associated with public funding of assisted reproduction. Finally, our results are only applicable to Spain.

In conclusion, an increase in public funding for assisted reproductive techniques in Spain might have positive effects in at least three respects. Notwithstanding the fact that general accounting is only a speculation, our results show that public funding of assisted reproduction might lead to positive net fiscal results for the Spanish Treasury during the life cycles of children conceived through assisted reproduction. Moreover, it would decrease the problem of lack of access to healthcare service, while most likely improving equity in access to the service and quality of life of infertile couples.
